# *Pseudomonas aeruginosa* pilin activates the inflammasome

**DOI:** 10.1111/j.1462-5822.2010.01541.x

**Published:** 2011-03

**Authors:** Cecilia S Lindestam Arlehamn, Tom J Evans

**Affiliations:** Institute of Infection, Immunity and Inflammation, College of Medicine, Veterinary and Life Sciences, University of GlasgowGlasgow, UK.

## Abstract

IL-1β is produced from inactive pro-IL-1β by activation of caspase-1 brought about by a multi-subunit protein platform called the inflammasome. Many bacteria can trigger inflammasome activity through flagellin activation of the host protein NLRC4. However, strains of the common human pathogen *Pseudomonas aeruginosa* lacking flagellin can still activate the inflammasome. We set out to identify what non-flagellin components could produce this activation. Using mass spectroscopy, we identified an inflammasome-activating factor from *P. aeruginosa* as pilin, the major component of the type IV bacterial pilus. Purified pilin introduced into mouse macrophages by liposomal delivery activated caspase-1 and led to secretion of mature IL-1β, as did recombinant pilin purified from *Escherichia coli*. This was dependent on caspase-1 but not on the host inflammasome proteins NLRC4, NLRP3 or ASC. Mutants of *P. aeruginosa* strain PA103 lacking pilin did not activate the inflammasome following infection of macrophages with live bacteria. Type III secretion remained intact in the absence of pili, showing this was not due to a lack of effector delivery. Our observations show pilin is a novel activator of the inflammasome in addition to flagellin and the recently described PrgJ protein family, the basal body rod component of the type III apparatus.

## Introduction

IL-1β is a key inflammatory cytokine important in host defence that mediates a diverse range of effects, including T-cell polarization, antibody production, fever and endothelial and phagocyte activation (O'Neill, 2008; Dinarello, 2009). IL-1β is an ancient molecule, appearing early in vertebrate evolution, with clear homologues in teleost fish indicating conservation over 450 million years since fish and tetrapods split (Bird *et al*., 2002). IL-1β is synthesized as an inactive pro form that requires proteolytic processing to the mature active protein that is released from cells (Auron *et al*., 1984). This processing is carried out by caspase-1, itself produced as an inactive precursor that also requires proteolytic processing to become active (Thornberry *et al*., 1992). This is a tightly controlled process performed by a multi-subunit protein complex termed the inflammasome, located in the cytoplasm (Yu and Finlay, 2008; Franchi *et al*., 2009; Martinon *et al*., 2009). The inflammasome brings together a sensor protein and the inactive caspase-1 zymogen, usually with an adaptor protein. A variety of diverse stimuli can activate the inflammasome, leading to proteolytic processing of caspase-1, maturation of pro-IL-1β to IL-1β and its release from the cell. This is often accompanied by a form of cell death termed pyroptosis (Bergsbaken *et al*., 2009). Understanding the triggers that lead to inflammasome activation following microbial infection is crucial in appreciating how IL-1β innate host defence is controlled, how microbes might evade such detection and how this system might be manipulated to augment both innate and acquired immune responses.

The sensor protein within the inflammasome typically belongs to the nucleotide-binding domain leucine-rich repeat containing (NLR) gene family (Ting *et al*., 2008; Ye and Ting, 2008). Distinct inflammasomes are formed that are differentiated by the NLR protein that they contain, and that respond to distinct stimuli. NLRs form a large protein family with over 20 members in humans and mice. Two prototypic inflammasomes are those based on the NLR protein NLRP3 [also known as NALP3 or cryopyrin (Agostini *et al*., 2004; Mariathasan *et al*., 2006; Sutterwala *et al*., 2006)] and NLRC4 [also known as IPAF, CARD12 or CLAN (Mariathasan *et al*., 2004)]. The NLRP3 inflammasome is activated by a range of stimuli, including ATP, uric acid crystals, alum and pore-forming bacterial toxins (Martinon *et al*., 2009). In contrast, the NLRC4 inflammasome is activated by intracellular microbial products (Evans, 2009), the best characterized ofwhich is the bacterial protein flagellin, the main component of flagella (Franchi *et al*., 2006; Miao *et al*., 2006). Both NLRP3 and NLRC4 inflammasomes usually contain the adaptor protein ASC, which is also required for activation (Mariathasan *et al*., 2004).

Most Gram-negative bacteria that can activate the NLRC4 inflammasome do so in a way that is completely dependent on the bacterial type III secretion system (TTSS). The TTSS is a mechanism whereby Gram-negative bacteria can introduce a variety of virulence factors directly into the host cell (Cornelis and Van Gijsegem, 2000). Structurally, the TTSS resembles a hypodermic syringe: the ‘needle’ of the complex forms a conduit between the bacterium and the host cell cytoplasm through which a variety of toxins can be passed (Johnson *et al*., 2005). *Pseudomonas aeruginosa* is an important human pathogen that possesses a TTSS. It is the main cause of hospital-acquired pneumonia and of lung disease in patients with cystic fibrosis, as well as infections in patients with neutropenia and burns (Pier and Ramphal, 2005). *P. aeruginosa* produces strong activation of the NLRC4 inflammasome in a TTSS-dependent fashion (Franchi *et al*., 2007; Sutterwala *et al*., 2007; Miao *et al*., 2008); flagellin introduced into the host cell cytoplasm via the TTSS is suggested to be the means whereby this organism activates the NLRC4 inflammasome. However, non-flagellated strains of *P. aeruginosa* can activate the inflammasome, indicating that there must be other mechanisms by which the NLRC4 inflammasome can be activated (Sutterwala *et al*., 2007).

Here, we set out to discover what other microbial factors from *P. aeruginosa* could activate the inflammasome. We have identified bacterial pilin, the main protein constituent of bacterial type IV pili as a novel inflammasome activator when introduced into macrophage cytoplasm. This is delivered in a process dependent on the bacterial TTSS, probably via the secretion apparatus itself. Type IV pili are found in a variety of different bacteria with conservation of the pilin molecule and are often important virulence determinants (Craig *et al*., 2004), all features that are typical of microbial determinants recognized by innate immune receptors. While this work was ongoing, the PrgJ family of proteins were identified as activators of the NLRC4 inflammasome (Miao *et al*., 2010). Pilin activation of the inflammasome provides an additional innate immune mechanism that can recognize pathogenic bacteria and initiate a powerful host defence response.

## Results

### A pseudomonal protein distinct from flagellin activates the inflammasome

**Fig. 1:**
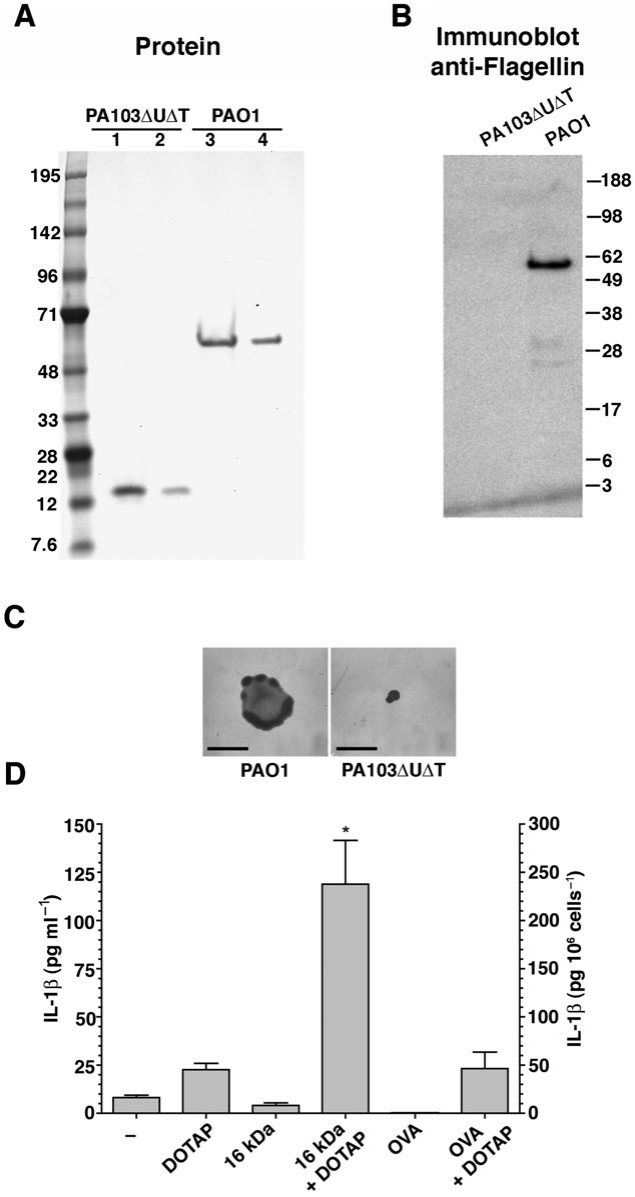
A 16 kDa protein fraction from *P. aeruginosa* PA103 can activate the inflammasome. A. SDS-PAGE analysis of material purified after agitation of indicated bacterial suspension cultures and stained with colloidal Coomassie Blue. 400 ng (lanes 1 and 3) or 200 ng (lanes 2 and 4) protein loaded. Molecular weight markers (kDa) shown to the left. Experiment repeated over five times. B. Immunoblot of same protein material as in a using anti-flagellin antibody. Experiment repeated with same results. C. Phenotypic analysis of presence of flagella using swimming motility plates for indicated bacteria. Bar shows 2 cm. Repeated more than three times with same results. D. IL-1β release following treatment of BMDMs as indicated. Values are corrected for release of unprocessed IL-1β. Left-hand *y*-axis shows absolute values obtained (pg ml^−1^); right-hand axis shows amount (pg) produced per 10^6^ cells. Columns are means of three independent determinations and same results obtained on more than three occasions; error bars are ± SEM. The asterisk indicates significantly different from DOTAP alone, 0.01 < *P* < 0.05, unpaired *t*-test. 16 kDa is the protein fraction shown in a lane 1, OVA is ovalbumen.

The *P. aeruginosa* strain PA103ΔUΔT has a competent type III secretion system but does not translocate any toxins (Vallis *et al*., 1999). This strain lacks flagellin (Liu, 1966) yet is still able to activate the inflammasome via the host NLR protein, NLRC4 (Sutterwala *et al*., 2007). This is dependent on the bacterial type III secretion system, but not on any secreted toxin. In an effort to identify the factor produced by PA103ΔUΔT responsible for activating the inflammasome, we purified material shed from the bacterium after physical agitation, using a protocol designed to isolate flagellin. Analysis of this material by SDS-PAGE showed it to contain a single detectable protein band of approximate mass 16 kDa (Fig. 1A). This was in contrast to protein isolated in the same fashion from the flagellated strain PAO1, which has molecular mass of approximately 53 kDa, as described for the b type flagellin of this strain (Arora *et al*., 2000). We confirmed this by immunoblotting this material with an antibody to flagellin, which recognized a 53 kDa protein in the PAO1 strain but did not react with material from PA103ΔUΔT (Fig. 1B). Flagellin-mediated swimming motility was also absent in PA103ΔUΔT but present in PAO1 (Fig. 1C).

We tested this 16 kDa protein containing fraction from PA103ΔUΔT for its ability to activate the inflammasome within bone-marrow-derived murine macrophages (BMDMs) in the presence of the liposomal transfection reagent DOTAP to introduce the protein directly into the macrophage cytoplasm. This combination resulted in secretion of mature IL-1β from BMDMs (Fig. 1D), whereas the protein alone or DOTAP alone produced little IL-1β. Control transfections with ovalbumen also failed to produce significant IL-1β (Fig. 1D). The levels achieved in our transfections with the 16 kDa material were over 200 pg 10^6^ cells^−1^ (Fig. 1D, right hand *y*-axis), which is similar to the levels observed from transfected purified flagellin (Miao *et al*., 2006 and C. S. Lindestam Arlehamn and T. J. Evans, unpubl. obs.).

### The 16 kDa protein from PA103ΔUΔT that activates the inflammasome is type IV pilin

The 16 kDa protein shown in Fig. 1A was isolated, digested with trypsin and individual peptides separated by liquid chromatography before sequencing using tandem mass spectrometry and analysis with the programme Mascot (Perkins *et al*., 1999) (Fig. S1). Nine unique peptides were sequenced and matched to the 15.8 kDa pilin protein from strain PA103 with a score of 830, corresponding to a probability of match by chance alone of 10^−83^. This protein accounted for 96% of the protein band analysed, as determined by the exponentially modified protein abundance index (Perkins *et al*., 1999).

**Fig. 2:**
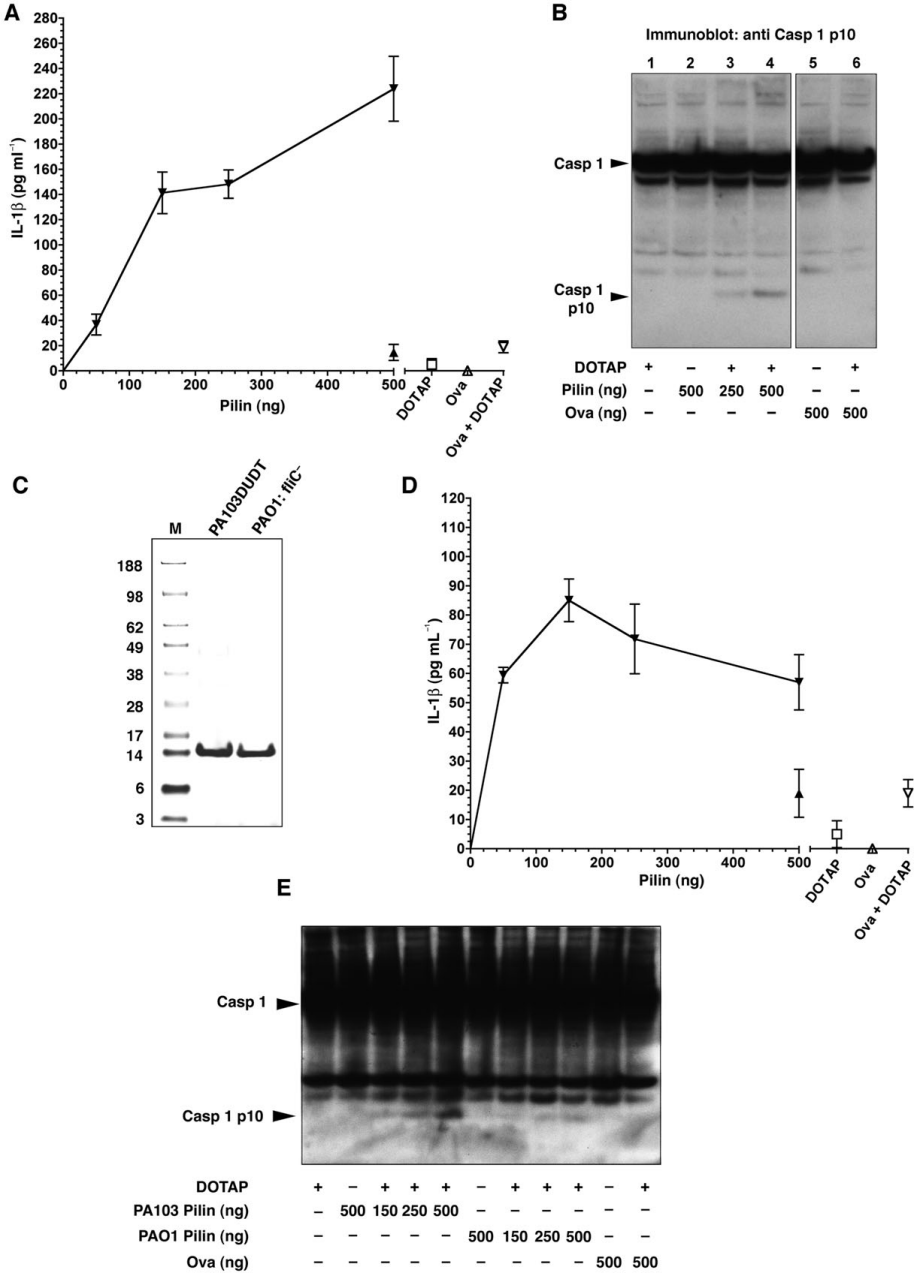
Dose–response curves of pilin activation of the inflammasome. A. Secreted IL-1β from BMDMs (corrected for release of unprocessed IL-1β) in response to indicated amounts of pilin in the presence of DOTAP (inverted filled triangles). Result with pilin alone at 500 ng shown as filled triangle. Values are means of three independent determinations; error bars are ± SEM. Repeated on two further occasions with similar results. Values for DOTAP alone, Ova and Ova plus DOTAP are shown to the right. Differences between doses of pilin are statistically significant (One-way ANOVA, *P* < 0.0001), with a significant increase with dose (*P* < 0.0001). B. Immunoblot with anti p10 caspase-1 of cell extracts from BMDMs treated as shown. Arrows indicate the unprocessed caspase-1 (Casp 1) and the p10 processed subunit (Casp 10 p10). These bands are absent in infected cells from caspase-1 knockout animals [(Lindestam Arlehamn *et al*., 2010) and data not shown]. Representative of three separate experiments. C. SDS-PAGE analysis of material purified after agitation of indicated bacterial suspension cultures and stained with colloidal Coomassie Blue. Molecular weight markers (kDa) are shown to the left. Representative of three separate experiments. D as A, but using pilin purified from PAO1:*fliC*^-^. Differences between doses of pilin are statistically significant (One-way ANOVA, *P* = 0.0001), with a significant increase with dose (*P* = 0.004). E. Immunoblot with anti p10 caspase-1 of cell extracts from BMDMs treated as shown. Arrows indicate the unprocessed caspase-1 (Casp 1) and the p10 processed subunit (Casp 10 p10). Experiment repeated with similar results.

To extend these observations, we re-purified material from PA103ΔUΔT using a protocol specific for isolation of pilin, and tested its ability to activate the inflammasome at different concentrations in combination with the transfection reagent DOTAP (Fig. 2A and B). We found as before that pilin with DOTAP stimulated the production of IL-1β, whereas pilin or DOTAP alone had no or little effect; the control protein ovalbumen plus DOTAP was also ineffective (Fig. 2A). In these experiments, cytotoxicity was < 15% and all IL-1β levels are corrected for release of unprocessed pro-IL-1β as described in *Methods*. Note also that LPS (2 µg ml^−1^) is continuously present in the experimental set-up, including when the DOTAP is added to the cells. However, in contrast to other reports (Kanneganti *et al*., 2007), we only found small amounts of IL-1β produced under these conditions, which were always significantly increased by the addition of pilin.

In a similar fashion, introduction of PA103ΔUΔT pilin into BMDMs produced activation of caspase-1, as evidenced by the appearance of the processed p10 subunit of caspase-1 following transfection of the pilin protein (Fig. 2B). Again, as a control, transfected ovalbumen was without effect, as was equivalent volumes of material purified form bacteria lacking pilin. Thus, introduction of pilin into the cytoplasm of BMDMs results in activation of the inflammasome, with processing of caspase-1 and production of mature IL-1β, but little cytotoxicity.

Next, we purified pilin from a different strain of *P. aeruginosa*, PAO1. This strain is normally flagellated (Fig. 1B and C). Using the same purification protocol as used for PA103ΔUΔT, we failed to isolate any pilin from the wild-type PAO1 strain (Fig. 1A), despite this strain having twitching motility, a phenotypic marker of functional type IV pili [(Murray and Kazmierczak, 2008) and data not shown]. However, when the flagellin gene *fliC* was deleted, pilin could be readily purified from the mutant (Fig. 2C). This suggests that in the presence of flagellin, either the level of pilin expression is significantly downregulated or that it becomes much more tightly associated with the bacterial cell and hence not amenable to biochemical purification. PAO1 pilin transfected into BMDMs activated the inflammasome leading to secretion of mature IL-1β and processing of caspase-1 (Fig. 2D and E). However, the absolute level of IL-1β released was less than with the PA103 pilin as was the degree of caspase-1 cleavage, suggesting that pilin from PA103 is a more potent activator of the inflammasome than that from PAO1.

**Fig. 3:**
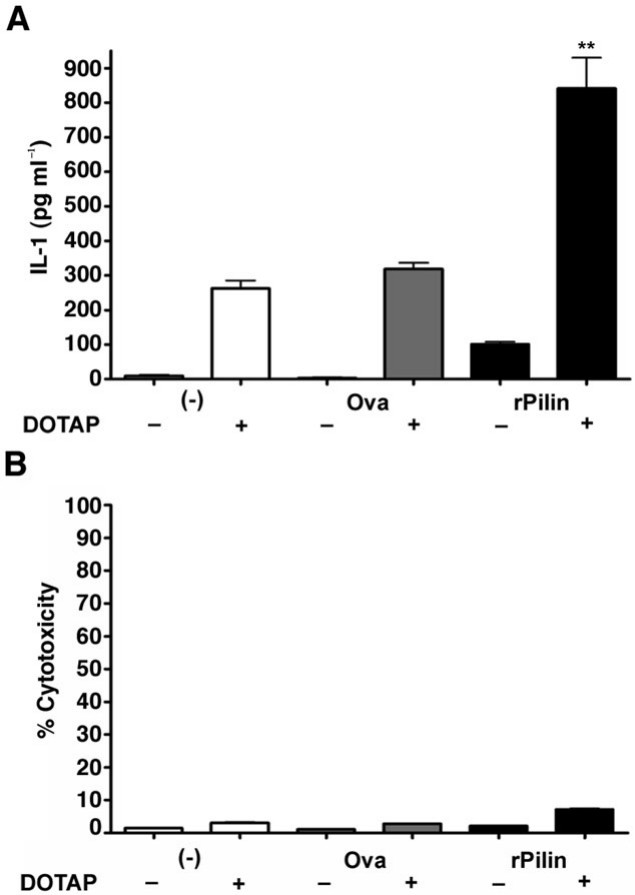
Recombinant pilin activates the inflammasome. Purified recombinant pilin (rPilin), ovalbumen or nothing was added to BMDMs in the presence or absence of the transfection reagent DOTAP as indicated. Cells were analysed for release of mature IL-1β (A) or cytotoxicity (B) as shown. rPilin produced a significantly higher amount of IL-1β compared with ovalbumen (**, two-sample *t*-test, *P* < 0.01). Results are the means of triplicate determinations; error bars are ± 1 SEM. The experiment was repeated on two further occasions with the same result.

To provide additional evidence that pilin is an activator of the inflammasome, we expressed PA103 pilin as a fusion protein in *Escherichia coli*, removed the fusion partner, and then purified the pilin away from other proteins, as described in *Methods* and shown in Fig. S2. Full-length pilin is very insoluble because of its hydrophobic N-terminus (Hazes *et al*., 2000). Thus, the expressed recombinant protein was truncated to omit the first 30 amino acids. When this protein was introduced into BMDMs using DOTAP, there was production of mature IL-1β with little cytotoxicity (Fig. 3). DOTAP alone produced rather more IL-1β than seen in Fig. 2; this varied with the batch of DOTAP and suggests additional mechanisms of inflammasome activation.

### Inflammasome components required to detect transfected pilin

**Fig. 4:**
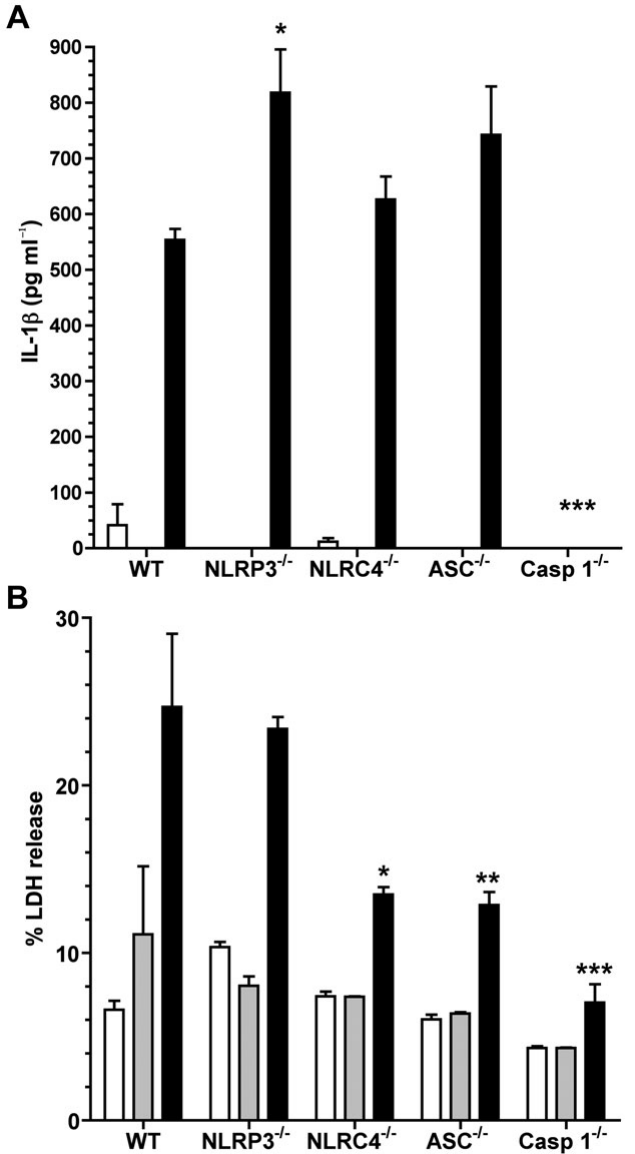
Pilin activation of the inflammasome is dependent on caspase-1 but not NLRP3, NLRC4 or ASC. BMDMs from wild-type (WT) mice or animals were transfected with DOTAP alone (open bars), pilin alone (grey bars) or DOTAP + Pilin (black bars). A shows secreted IL-1β corrected for release of immature pro-IL-1β. Bars are means of triplicate independent determinations; error bars are ± SEM. Similar results were obtained on repeating the experiment on two further occasions. There were significant differences between the values of pilin + DOTAP between the different animals (*P* < 0.0001 one-way ANOVA), with significant rise in the NLRP3^−/−^ mice (*P < 0.05 vs. WT animals, Bonferroni multiple comparison test) and highly significant reduction in Casp 1^−/−^ mice (***P < 0.001 vs. WT animals, Bonferroni multiple comparison test). B shows cytotoxicity after infection of the macrophages from the various mice strains as measured by LDH release. Bars are means of triplicate independent determinations; error bars are ± SEM. Differences between mice strains are significant (*P* = 0.005, one-way ANOVA) with significant reductions as tested by Bonferroni multiple comparison test in BMDMs from NLRC4^−/−^ mice (*P < 0.05), ASC^−/−^ mice (*P* < 0.01) and Casp 1^−/−^ mice (*P* < 0.001) all compared with WT.

A number of proteins play a role in inflammasome activation following infection of BMDMs with *P. aeruginosa*. NLRC4 is the key component identified as responsible for activation of macrophages by flagellin. The adaptor protein, ASC, also plays a role in this process. NLRP3 is important in inflammasome activation in response to ATP and a number of other diverse stimuli, but not in response to infection with *P. aeruginosa*. In order to determine which inflammasome components were required to respond to transfected pilin, we isolated BMDMs from mice with homozygous deletions in these genes, as well as caspase-1, the enzyme responsible for IL-1β processing. Using these cells, we then assayed their ability to produce IL-1β following transfection of PA103 pilin with DOTAP (Fig. 4). As before, wild-type BMDMs responded to pilin only in the presence of the transfection reagent DOTAP. Cells from animals with homozygous deletion of NLRC4, NLRP3 and ASC responded to transfected pilin as well as wild-type cells (Fig. 4). Indeed, cells from the NLRP3^−/−^ mice produced a just significantly higher amount of IL-1β than the wild type. Cells from mice with knockout of caspase-1 did not produce any response. Thus, transfected pilin activates the inflammasome in a fashion that is not dependent on NLRC4, NLRP3 or ASC but does depend on caspase-1. This is in contrast to the components required to respond to PA103 infection, which is dependent on NLRC4 and ASC as well as caspase-1. This is considered further in *Discussion*.

We also measured cytotoxicity in these experiments as % LDH release. In the wild-type mice in this experiment, we observed rather higher cytotoxicity of about 25% compared with the experiments reported earlier with transfected pilin (Fig. 4B). This variability seemed to correlate with the batch of DOTAP being used. Cells from mice with knockout of NLRC4 and ASC did show a significant reduction in cytotoxicity compared with wild-type cells, suggesting these components contribute to cytotoxicity if not IL-1β production (Fig. 4B).

### Pilin activation of the inflammasome is protein and not DNA dependent

**Fig. 5:**
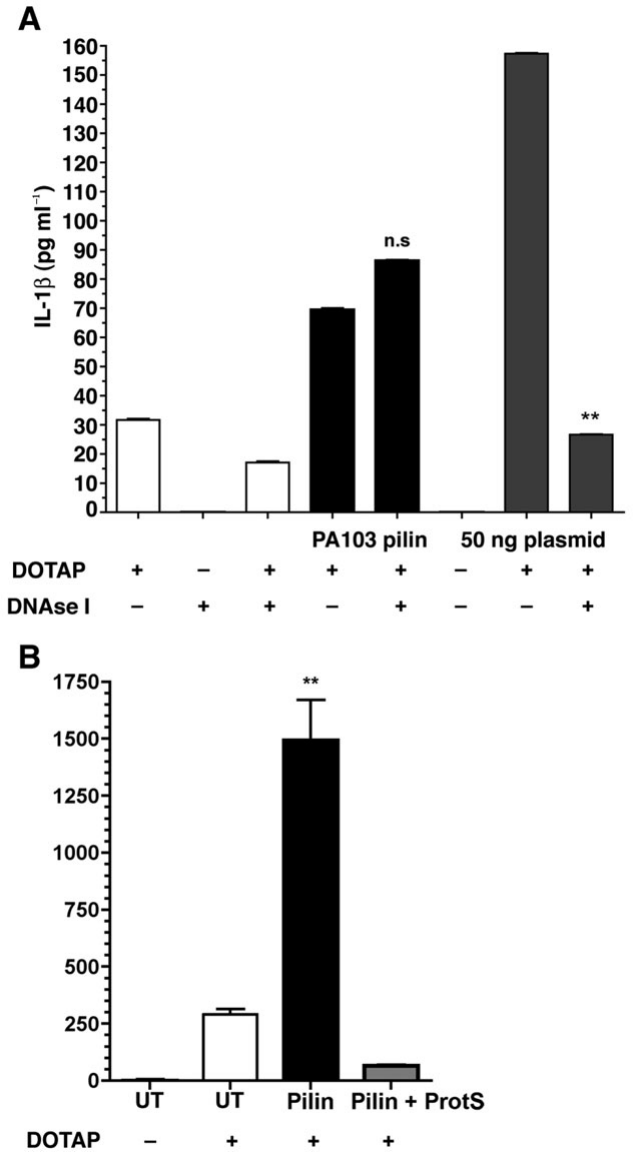
Pilin stimulation of the inflammasome is not affected by DNAse and is reduced by pyrococcal protease S. A. BMDMs were treated as indicated with PA103 pilin (250 ng) or plasmid DNA (pUCP20) and DOTAP and/or DNAse I as shown prior to IL-1β measurement in supernatant. Bars are means of three independent determinations; errors bars are ± SEM. Effects of DNAse I treatment were compared with control values by unpaired *t*-test. For pilin no significant change was observed (n.s. not significant); DNAse significantly reduced the IL-1β released (**P < 0.001). B. Pilin was left untreated or digested with pyrococcal protease S before stimulation of BMDMs in the presence of DOTAP and measurement of released IL-1β. Results are means of three independent determinations; error bars are ± SEM. Untreated pilin + DOTAP produced significantly more IL-1β than DOTAP alone (**P < 0.001, unpaired *t*-test); proteinase S-treated material was not significantly different from control.

Bacterial DNA is known to activate the inflammasome following transfection and might contaminate the protein samples used in these experiments, although transfected DNA has a strict requirement for ASC to activate the inflammasome, so this would seem unlikely. To be certain this was not the case, we assayed for DNA in our samples and found it was less than the limit of detectability (< 5 ng). As a further control we subjected the pilin sample to DNAse I digestion. This did not alter its ability to activate the inflammasome (Fig. 5A). Control digests of plasmid DNA confirmed that the DNAse was active and could abolish the inflammasome activating activity of plasmid DNA (Fig. 5A). Additionally, pilin did not inhibit the ability of DNAse I to digest DNA (Fig. S3).

As a further confirmation that the protein pilin was responsible for inflammasome activation following transfection, we digested it with pyrococcal protease S. This significantly reduced its ability to activate the inflammasome to the levels seen with the DOTAP transfection agent alone (Fig. 5B). This supports the conclusion that the material purified from bacteria that activates the inflammasome is protein.

### Pilin mutants abolish inflammasome activation by PA103

**Fig. 6:**
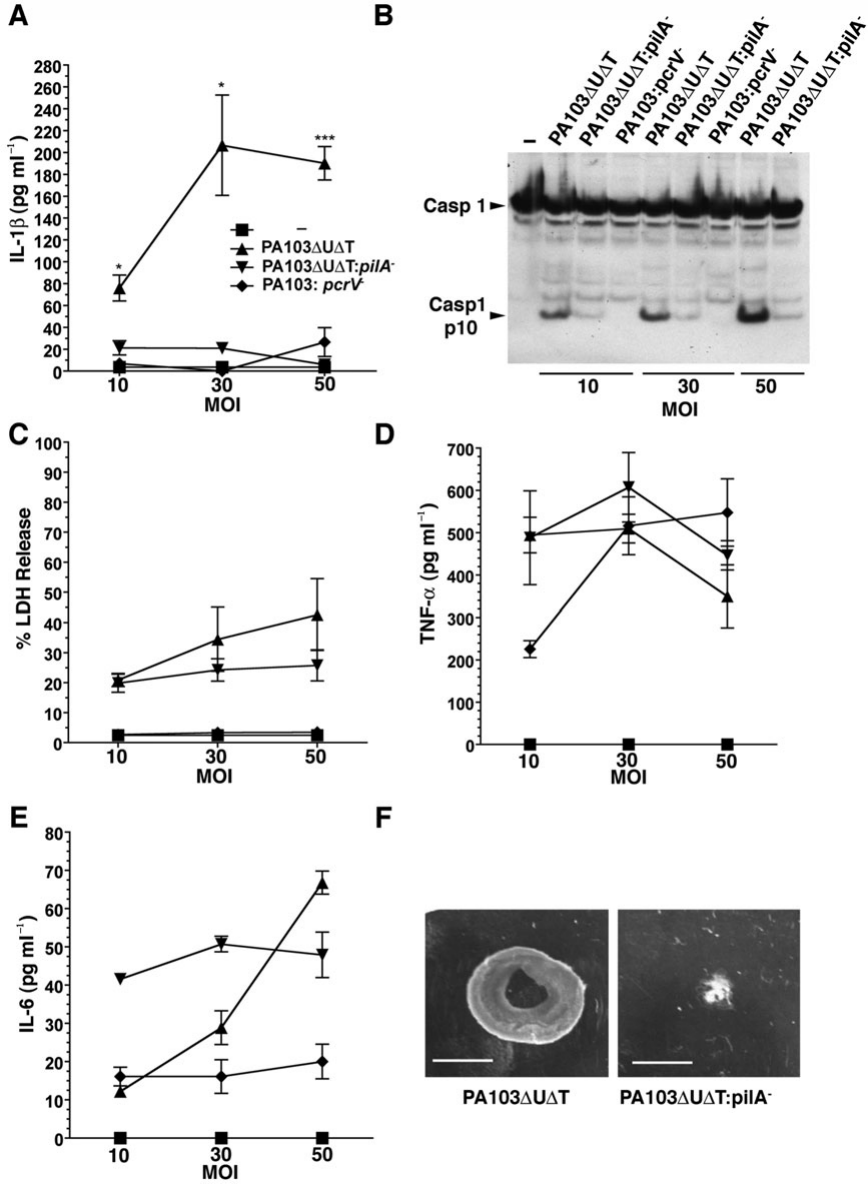
Loss of pilin reduces activation of the inflammasome following infection of BMDMs with *P. aeruginosa* strain PA103. BMDMs were infected with PA103ΔUΔT, PA103*pcrV*^-^ or PA103ΔUΔT: *pilA*^-^ at different MOIs or left uninfected. Following infection, secreted IL-1β (A), TNF-α (D) and IL-6 (E) were determined as well as released LDH as a marker of cytotoxicity (C). Each point is the mean of three separate determinations; error bars are ± SEM. Differences between the IL-1β produced by PA103ΔUΔT and PA103ΔUΔT: *pilA*^-^ strains were significant at all MOIs (unpaired *t*-test, *P < 0.05, ***P < 0.001). B shows cellular lysates of BMDMs infected as shown and immunoblotted for the p10 activated caspase-1 fragment. Arrows to the left indicate unprocessed caspase-1 (Casp 1) and the p10 fragment (Casp1 p10). F shows pilin-dependent twitching motility of the PA103ΔUΔT strain that is absent when the *pilA* gene is deleted, PA103ΔUΔT: *pilA*^-^. Bar is 2 cm. All experiments were repeated at least once.

Next, we tested strains of PA103 lacking the gene encoding pilin (*pilA*) for their ability to activate the inflammasome. At a number of different multiplicities of infection (MOI) the strain lacking pilin (PA103 ΔUΔT *pilA*^-^) failed to produce IL-1β secretion (Fig. 6A). IL-1β secretion by PA103 ΔUΔT *pilA*^-^ was comparable with the PA103 ΔpcrV mutant that does not elaborate a functional type III apparatus (Fig. 6A). Caspase-1 processing by the *pilA*^-^ mutant was considerably reduced at all MOIs compared with the wild-type strain (Fig. 6B) although not to the same extent as the PA103 ΔpcrV mutant. Cytotoxicity as measured by LDH release was not significantly different between PA103 ΔUΔT wild type and *pilA*^-^ strain at all the MOIs tested (Fig. 6C); the PA103 ΔpcrV strain had lower cytotoxic effects that did not significantly differ from the values for uninfected cells (Fig. 6C). All three strains were able to induce macrophage production of TNF-α, with no significant difference between them (Fig. 6D). IL-6 production after macrophage infection was higher for the *pilA*^-^ strain compared with the *pcrV*^-^ mutant at all MOIs, while that for the wild-type strain increased with increasing MOI (Fig. 6E). Functional loss of pilin was confirmed in the *pilA*^-^ mutant by assaying for twitching motility (Fig. 6F), a characteristic property of type IV pili (Mattick, 2002). Taken together, these data show that inflammasome activation by PA103 ΔUΔT is dependent on the presence of type IV pili.

### Lack of pilin does not impair type III toxin translocation into macrophages

**Fig. 7:**
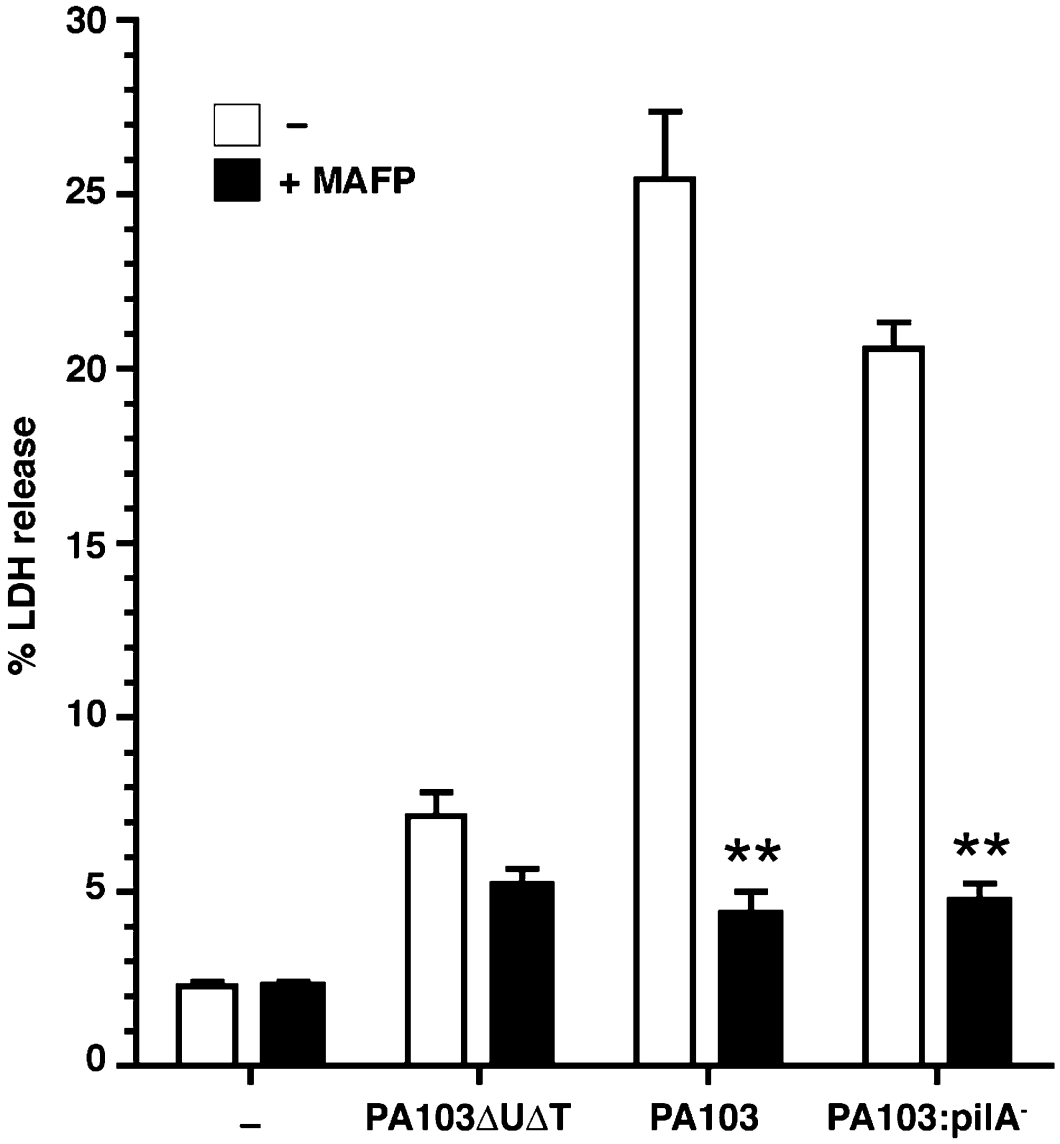
Type III translocation of ExoU into BMDMs is not defective in *pilA*^-^ mutants. BMDMs were infected with the ExoU secreting strains PA103 (wild type) and PA103: *pilA*^-^ as well as the non-ExoU translocating strain PA103 ΔUΔT, all at MOI of 30. Graph shows the cytotoxicity following infection with the indicated strains measured as % release of LDH in the absence (open bars) or presence (filled bars) of the ExoU inhibitor MAFP. Bars are means of three independent determinations; error bars are ± SEM. There was no statistically significant difference between the PA103 and PA103: *pilA*^-^ strains in the cytotoxicity they produced. In the presence of MAFP, both strains showed a statistically significant reduction in cytotoxicity compared with absence of inhibitor (**P < 0.01, unpaired *t*-test). The experiment was repeated on two further occasions with the same results.

Type IV pili are known to act as adhesins for *P. aeruginosa*, mediating attachment to epithelial cells (Hahn, 1997). Thus, one possible explanation for the loss of inflammasome activation observed in the PA103ΔUΔT*pilA*^-^ mutant is that it fails to attach to macrophages and cannot mediate type III secretion, hence behaving like the *pcrV*^-^ mutant, which cannot activate the inflammasome. To investigate the ability of the *pilA*^-^ mutant to translocate a type III secreted effector, we assayed for the translocation of the type III exported toxin ExoU, a potent phospholipase (Sato *et al*., 2003). This toxin produces significant cytotoxicity when translocated into host cells that can be abrogated by inhibiting its phospholipase activity with MAFP (Sato *et al*., 2003), but is completely inactive when extracellular as it has an absolute requirement for an intracellular eukaryotic cofactor now identified as superoxide dismutase (Sato *et al*., 2006). Pseudomonal strains lacking the essential TTSS component PcrV cannot translocate toxins into cells but constitutively secrete exotoxins into the growth media (Sawa *et al*., 1999). *PcrV* mutants of ExoU secreting Pseudomonal strains are not cytotoxic, confirming that ExoU cannot act extracellularly (Sawa *et al*., 1999). The PA103ΔUΔT strain gives little cytotoxicity following infection of BMDMs as noted before (Fig. 7). The wild-type PA103 produces considerable cytotoxicity that is abolished by treatment with the ExoU inhibitor MAFP (Fig. 7). This reflects its ability to translocate ExoU into the macrophages. The cytotoxic effect of PA103 lacking *pilA* (PA103 *pilA*^-^), but still encoding a functional ExoU protein, was not significantly different from the wild-type strain. Importantly, this cytotoxic action could be abolished by treatment with the ExoU phospholipase inhibitor MAFP (Fig. 7). Thus, lack of pili did not prevent *P. aeruginosa* translocating ExoU into cells, showing that pili are not required for effective type III translocation into macrophages.

## Discussion

The data presented here have identified type IV pilin from *P. aeruginosa*, the major structural protein of type IV pili, as a novel activator of the inflammasome when introduced into the cytoplasm via the bacterial TTSS. Both natural and recombinant pilin activated the inflammasome when transfected into cells (Figs 1–3) and the activity was protease-sensitive but not destroyed by DNase (Fig. 5). The PA103 strain lacking pilin was unable to activate the inflammasome despite continued translocation of type III exported toxins (Fig. 7). Taken together, these data demonstrate that both biochemically and in an *in vitro* infection model, translocation of pilin via the type III secretion system of *P. aeruginosa* can activate the inflammasome. This is the first demonstration of recognition of type IV pilin by pattern recognition receptors of the innate immune system.

In almost all species, type IV pili are important virulence determinants (Craig *et al*., 2004). Structurally, they are formed of thousands of copies of pilin that show sequence similarities between different species. These are all attributes that would engender a strong selection pressure for a pilin-specific pattern recognition receptor as described here. Two main type IV pilin subtypes are recognized: type IVa and IVb that differ in length, leader sequence and N-terminal modification (Craig *et al*., 2004). *P. aeruginosa* pilins belong to the type IVa group and have primary amino acid sequence homology to other type IVa pilins. There is, however, some variation between different strains of *P. aeruginosa*, with PA103 pilin having 80% identity with PAO1 pilin (GenBank Accession AAG07913) but only 65% identity with pilin from the PAK strain (GenBank Accession P02973). A diagram showing the sequence similarities between pilin from the pseudomonal strains PAO1 and PA103 is shown in Fig. S4. Additionally, *P. aeruginosa* has five different phylogenetic types of *pilA* alleles, with differences in post-translational modifications and different associations with cystic fibrosis (Kus *et al*., 2004). Such variability in pilin sequence can contribute to evading both innate and acquired immune responses. Sequence variations in flagellin between different bacterial species have also been associated with loss of recognition by an innate pattern recognition receptor, TLR5 (Andersen-Nissen *et al*., 2005). The differences reported here between the potency of PA103 and PA01 pilin in activating the inflammasome may reflect such selection pressure. Glycosylation of pilin may also differ between strains and contribute to variability in inflammasome response (Smedley *et al*., 2005).

Structural analysis of pilin shows a high degree of architectural similarity between pilin from different bacterial species. All pilins possess an N-terminal hydrophobic α-helix that mediates pilus assembly by packing into a hydrophobic helical bundle in the core of the filament (Craig *et al*., 2004). This region shows the greatest degree of amino acid conservation between pilins from different organisms. The main areas of the molecule that are exposed in the complete fibre are the area between the N-terminal α-helix and the β sheets of the globular head domain, and the C-terminal D region that contains a conserved disulphide bridge. Given that a recombinant truncated pilin lacking the N-terminal hydrophobic domain can activate the inflammasome (Fig. 3), this region of the molecule does not appear to be required for inflammasome activation. The exposed D region of pseudomonal pilin is proposed to mediate attachment to epithelia through binding to the glycolipids asialo-GM1 and asialo-GM2 (Craig *et al*., 2004). However, this has been contradicted by other studies (Schroeder *et al*., 2001). Delineation of the region of the pilin molecule required to activate the inflammasome will require further work. The retroviral expression system employed by Lightfield *et al*. (Lightfield *et al*., 2008) is a powerful method to identify which regions of a protein are required for cell death as a marker of inflammasome activation. Although retroviral expression of pilin within BMDMs produced far fewer transduced cells, this activity was not dependent on the inflammasome (data not shown). Pilin must possess toxic activities that are independent of inflammasome activity and thus preventing this assay being used to follow inflammasome activation.

A number of groups initially reported that *P. aeruginosa* could activate the NLRC4 inflammasome in a process dependent on the TTSS (Franchi *et al*., 2007; Sutterwala *et al*., 2007; Miao *et al*., 2008). Miao *et al*. (Miao *et al*., 2008) ascribed this activation to the delivery of flagellin via the TTSS, although their earlier work in *Salmonella typhimurium* noted that at higher MOIs there was a flagellin-independent mechanism leading to activation of the NLRCP3 inflammasome. Sutterwala *et al*. noted that *P. aeruginosa* strains lacking flagellin could still activate the inflammasome, which they felt most likely was due to a ‘pore-like’ effect produced by the TTSS apparatus rather than a specific product delivered into the host cell cytoplasm. Miao *et al*. subsequently have discovered that the TTSS PrgJ rod protein family can also activate the inflammasome (Miao *et al*., 2010). The PscI protein of *P. aeruginosa* is a member of this protein family and can produce NLRC4 activation. Our work identifies another pseudomonal protein, pilin, that can activate the inflammasome. What relative role do these different bacterial proteins – flagellin, PscI and pilin – play in activating the inflammasome in the course of an infection with *P. aeruginosa?*

Although flagellin is widely expressed among pathogenic bacteria, there are many examples where flagella are not produced by bacteria that produce disease, *P. aeruginosa* included (von Götz *et al*., 2004). Thus, the ability of pilin to activate the inflammasome as described here can play an additional role in host defence against a range of bacteria. Further research will be required to determine the extent to which pilins from different bacterial species are able to induce inflammasome activation. Equally, the relative role of the type III rod protein PscI in activating the inflammasome compared with either flagellin or pilin is not clear. How and under what conditions this protein is translocated by the type III apparatus is not known. Importantly, we do not feel that activation of the inflammasome by flagellin, PrgJ and pilin is mutually exclusive, but rather that they can all play a role in different bacterial strains and infection conditions. Further work is required to establish the relative contributions of these different pseudomonal proteins in activation of the inflammasome.

Although when pilin is introduced via the TTSS of the bacterium inflammasome activation is dependent on the components NLRC4 and ASC, when transfected into cells nether of these proteins was required, although the activation was caspase-1-dependent. Inflammasome activation by viral DNA also shows a differential requirement for inflammasome components depending on the means whereby the DNA reaches the cell cytoplasm (Muruve *et al*., 2008). Thus, adenovirus DNA introduced by infection of macrophages by virus activates the inflammasome by a NLRP3- and ASC-dependent process. However, when adenoviral DNA is introduced into cells by liposomal-mediated transfection, the inflammasome activation produced is not dependent on NLRP3, although it does remain ASC-dependent (Muruve *et al*., 2008). These data suggest that the sensors involved in detecting cytoplasmic danger signals such as DNA or pilin are compartmentalized, and that different inflammasome components may be utilized in different cellular compartments. As yet, no direct interaction has been detected between a pathogen-associated molecule and an inflammasome component, suggesting that either additional inflammasome components are involved, or that the activation is indirect. One possible means of indirect recognition is a system that monitors perturbation of a host protein by pathogen-derived molecules, as has been found in a number of examples of plant resistance that also uses NLR family proteins, the so-called ‘Guard Hypothesis’ (Marathe and Dinesh-Kumar, 2003). Further work will be required to identify the possible additional inflammasome components that may be involved in inflammasome activation by pilin. ASC-independent IL-1β production is described in *Legionella pneumophila* infection (Case *et al*., 2009) as well as activation of the NLRP1b inflammasome by muramyl dipeptide (Hsu *et al*., 2008).

As with delivery of flagellin into the host cell, inflammasome activation by pseudomonal pilin is dependent on the bacterial TTSS. Delivery of proteins via the TTSS is usually very specific, with secreted toxins requiring a specific protein chaperone to enter the TTSS apparatus. Because flagellin is exported into the flagellum via a TTSS, delivery into a host cell was proposed to occur through flexibility in the TTSS secretion signal (Miao *et al*., 2006). Type IV pili biogenesis requires the passage of pilin across bacterial membranes, a process involving over a dozen proteins (Proft and Baker, 2009). Passage of pilin across the outer membrane of Gram-negative bacteria requires a transmembrane protein of the secretin family (Bitter, 2003). Secretin family members also form the outer membrane part of the TTSS. We do not know how pilin reaches the host cell cytoplasm in a TTSS-dependent fashion, but one possible explanation is through recognition of pilin by secretin family members, resulting in ‘accidental’ passage of pilin through the TTSS. It has also been proposed that the TTSS may act as a somewhat leaky ‘pore’, enabling other bacterially derived macromolecules access to host cell cytoplasm in a non-specific fashion (Neyt and Cornelis, 1999). We find no evidence that the TTSS of *P. aeruginosa* forms a leaky pore (Lindestam Arlehamn *et al*., 2010). As with detection of flagellin, host recognition of cytoplasmic pilin is a sensitive indicator of bacterial invasion, a danger signal whose recognition by the innate immune system would provide considerable evolutionary selection advantage. Pilin may also be delivered into the host cell cytoplasm by escape from bacteria within the phagocytic vacuole. Whatever the mechanism, inflammasome activation and the production of IL-1β results in an appropriate immune response to invading bacteria, to augment the responses triggered by other bacterial components.

## Experimental procedures

### Bone-marrow-derived macrophages and mice

All animal studies were carried out under the relevant national guidelines. C57Bl/6 mice femurs and tibias were used a source of bone-marrow-derived macrophages. Bone-marrow mononuclear phagocytic precursor cells were seeded into untreated 9 cm Petri dishes (Sterilin) at a concentration of 3 × 10^6^ cells plate^−1^. Complete media were further supplemented with M-CSF from supernatant of L929 cells or as purified recombinant protein (10 ng ml^−1^, Peprotech). Cells were cultured for 6–7 days until mature, then scraped off and seeded into tissue culture dishes before experiments.

Where indicated, BMDMs were derived from mice with lack of caspase-1, NLRP3, NLRC4, and ASC, on a C57Bl/6 background as previously described (Mariathasan *et al*., 2004; Martinon *et al*., 2006).

### Bacterial strains and growth

PA103ΔUΔT and PA103*pcrV*^-^ were kind gifts from Dara Frank, Medical College of Wisconsin. PA103*pilA*^-^ and PAO1 were a kind gift from Barbara Kazmierczak, Yale University School of Medicine (Murray and Kazmierczak, 2008). PA103ΔUΔT*pilA*^-^ was constructed using standard methods from the strain PA103*pilA*^-^ (Choi *et al*., 2006).

Bacteria were grown routinely in Luria–Bertani (LB) media (Invitrogen) containing the appropriate antibiotics depending on bacterial strain. An overnight culture was diluted 1 in 30 in LB and allowed to grow for another 90 min or until OD600 was between 0.4 and 0.6. The bacteria were then centrifuged at 3000 *g* for 10 min at 4°C, the pellet was washed twice in sterile PBS and then resuspended in the same media as the cells being infected to a concentration of approximately 1 × 10^6^ cfu µl^−1^, by using a volume of (OD600/0.4) × 1.8 ml. Cells were then infected at the indicated MOI and time. When using mutants deficient in flagellin and/or pilin, bacteria were spun onto cells for 5 min at 1500 *g*; however, we did not find this made any significant difference to the amount of IL-1β released. In some experiments Methyl Arachidonyl Fluorophosphonate (MAFP; Calbiochem) was added at a final concentration of 100 µM 60 min before infection.

### Lactate dehydrogenase (LDH) assay

Lactate dehydrogenase release determinations were performed using the CytoTox 96 cytotoxicity assay kit (Promega). As a positive control for total cell-associated LDH, cells were lysed with 1% Triton X-100 at 37°C for 20 min. The % cell death was determined according to % cytotoxicity = (experimental LDH release/maximum LDH release) × 100.

### Western blotting

Following infection or stimulation as indicated, supernatants were kept for ELISA and LDH assays. The cells were washed in PBS and lysed in lysis buffer [50 mM Tris-HCl (pH 7.4)], 150 mM NaCl, 5 mM EDTA, 1% (v/v) Triton X-100 supplemented with a EDTA-free protease inhibitor cocktail (Complete Mini EDTA-free; Roche). After lysis for 15 min at RT cells were scraped off and stored at −70°C before Western blot analysis. For IL-1β analysis, blots were incubated with a rabbit polyclonal antibody to the p10 fragment of activated murine caspase-1 (Santa Cruz Biotechnology). Anti-flagellin antibody was a mouse monoclonal (Biolegend).

### ELISA for IL-1β

ELISA for IL-1β was performed according to manufacturer's instructions (BD Biosciences). The release of pro-IL-1β from dead cells was controlled for by analysing 0.9% Triton X-100 treated unstimulated macrophages, to release total pro-IL1β from within the cells. This can be detected by the ELISA, although the assay is more sensitive for the mature form. As described by Miao *et al*. (Miao *et al*., 2006), values from experimental wells were then corrected for the amount of pro-IL-1β released from the dead cells according to the formula: Mature IL-1β = total IL-1β signal − (pro-IL-1β lysis value × percent release of LDH). Each sample was assayed in duplicate. Results shown are the means of three separate experiments.

### Protein transfection

BMDM were seeded 2.5 × 10^5^ cells per well into 24-well plates (Corning) in complete media with 2 µg ml^−1^ LPS. After 3 h at 37°C in a humidified incubator cells were washed and media was replaced with 300 µl RPMI without Phenol Red (Invitrogen) supplemented with 2 mM L-glutamine and 2 µg ml^−1^ LPS. Proteins (at the indicated final concentrations) were diluted in media to a final volume of 50 µl and 2 µg N-[1-(2,3-Dioleoyloxy)propyl]-N,N,N-trimethyl-ammonium methylsulfate (DOTAP) liposomal transfection reagent (Roche) was added to appropriate tubes. After 15 min incubation at RT the transfection mixture was added to the cells and plates swirled to allow for even distribution. Plates were incubated at 37°C for 3.5 h or as indicated. Following this incubation cell lysates were prepared as described and supernatants were kept and analysed for LDH release and IL-1β secretion. Levels of IL-1β did show some variation between different batches of DOTAP, but in all cases simultaneous controls of protein alone and DOTAP alone were performed to allow comparison with the value for protein plus DOTAP. Note that LPS is continually present in our experimental set-up but this produced little IL-1β in combination with DOTAP and levels with pilin were always significantly higher as indicated.

### Pilin purification

The pilin purification method used was based on a method described by Smedley *et al*. (Smedley *et al*., 2005). Bacterial strains were grown in Trypticase Soy Broth (TSB) with very gently agitation (60 r.p.m.) at 37°C over night. The over night culture was pelleted at 8000 r.p.m. for 13 min at 4°C in a Beckman Coulter Avanti J-26 XP centrifuge. The pellet was resuspended in 25 ml PBS and vortexed hard, which was followed by vigorous stirring using a magnetic stirrer for 30 min at RT with intermediate vortexing. The bacterial suspension was then centrifuged at 16 000 r.p.m. in a Beckman Coulter Avanti J-26 XP centrifuge at 4°C for 30 min. The supernatant was kept and the NaCl concentration was increased to 0.5 M and polyethylene glycol PEG 3000 was added to a final concentration of 3% v/v. The solution was kept at 4°C for at least 18 h, following which precipitated proteins were collected by centrifugation at 11 000 *g* at 4°C for 20 min. The proteins were dissolved in 1 ml water and analysed using SDS PAGE. For protein visualization, gels were stained with colloidal Coomassie Blue (Instant Blue, Expedeon), which is able to detect 5 ng of protein per lane. Protein concentrations were determined using a modified Bradford assay (Bio-Rad) with BSA as a standard.

### Recombinant pilin production

An N-terminal truncated version of PA103 pilin was amplified by PCR using the primers gatggatccgcttcggaaggcgcttcggc (forward) and tcgaagcttggaatcgaacccccgacc (reverse) and cloned between the BamH1 and HindIII sites (underlined) of pET41a (Novagen, Merck Biosciences), which results in the pilin being expressed from amino acid 31 of the mature protein in frame with GST containing a His tag. The fusion protein was induced in Rosetta *E. coli* (Novagen, Merck Biosciences) according to the manufacturer's instructions. The fusion protein was purified from cleared bacterial lysates using nickel-NTA agarose (Invitrogen) according to the manufacturer's instructions. The GST tag was cleaved from the fusion protein using high activity recombinant enterokinase (Novagen, Merck Biosciences) and His tag bearing impurities removed by Ni-NTA agarose as before. The purity of the final protein was checked by SDS polyacrylamide gel electrophoresis (Fig. S2).

### DNAse and protease digestion

0.5 units of Pyrococcal protease S (Sigma) was added to samples and incubated at 60°C for 60 min. DNAse1 (Qiagen; 0.5 Kunitz units) was added to samples and incubation performed at 37°C for 30 min.

### Twitch and swimming assays

Type IV pilus-mediated twitching motility assays were performed as described (Semmler *et al*., 1999). Colonies from an agar plate were picked up with a p200 pipette tip and stabbed through the agar of a twitch agar plate. Plates were left at 30°C for 2 days and then analysed for the twitch zones between the bottom of the agar and the Petri dish. This was best visualized by careful removal of the agar and viewing with an oblique light source. Flagellin-mediated motility (‘swimming’) was determined as described.

### Statistical tests

Comparisons between two groups were made using the unpaired *t*-test. For more than two groups, one-way ANOVA was used, with post tests for trend or correction (Bonferroni) for multiple comparisons. Results were considered statistically significant if *P* < 0.05.

## Supporting information

**Fig. S1.** Peptides identified from mass spectroscopy of tryptic digest of 16 kDa protein matched to pilin using the programme MASCOT. Mr (expt) is experimental mass, Mr (calc) is calculated mass.

**Fig. S2.** Purification of recombinant truncated pilin. SDS poly-acrylamide gel showing protein fractions of the purified fusion protein (Fusion), the fusion protein after enterokinase digestion (Fusion + EK), and the final purified pilin after contaminant removal (Purified rPilin). Molecular weight markers are in lane M with values in kDa shown to the left.

**Fig. S3.** Addition of pilin does not inhibit DNAse I. Plasmid DNA was present in all samples and then digested with DNAse I as indicated in the presence or absence of pilin (250 ng). DNA was analysed by agarose gel electrophoresis and the presence of plasmid DNA was detected by ethidium bromide staining. Size markers (kb) are shown to the left.

**Fig. S4.** Diagram showing the sequence conservation between pilin from the pseudomonal strains PAO1 and PA103. The upper section shows the regions of identity between the two proteins as shaded gray; differences in sequence are left white. Numbering of the amino acids is shown above the blocks; 1 is the first amino acid of the mature protein i.e. after cleavage of the signal sequence. The lower section shows the main architectural features of the pilin molecule. The hydrophobic segment of the α1 helical segment is shaded dark gray. The α/β loop is shaded green and the D region shaded magenta, flanked by the two conserved cysteine residues, shaded yellow.
