# Supplementary material for: Pseudomonas aeruginosa pilin activates the inflammasome
Source: Cell Microbiol. 2011 Mar;13(3):388–401. doi: 10.1111/j.1462-5822.2010.01541.x (PMC3429865; doi:10.1111/j.1462-5822.2010.01541.x)
Supplement: Supplementary file 1 [file cmi0013-0388-SD1.pdf]

Mascot Search Results

**User**	: **Kenneth Beattie**
**Email**	: **k.a.beattie@dundee.ac.uk**
**Search title**	: **Submitted from Cecilia Lindestam NCBIall 230408 by Mascot Daemon on LS17985 (dUF**
**MS data file**	: **D:\Raw QTrap data\2008_04_23\dUFr.wiff**
**Database**	: **NCBInr (4565699 sequences; 1571958806 residues)**
**Timestamp**	: **24 Apr 2008 at 08:37:15 GMT**
**Enzyme**	: **Trypsin/P**
**Variable modifications**	: **Acetyl (N-term),Carbamidomethyl (C),Dioxidation (M),Gln->pyro-Glu (N-term Q),Oxi**
**Mass values**	: **Monoisotopic**
**Protein Mass**	: **Unrestricted**
**Peptide Mass Tolerance**	: **Â± 1.5 Da (# ^{13}C = 2)**
**Fragment Mass Tolerance**	: **Â± 0.5 Da**
**Max Missed Cleavages**	: **2**
**Instrument type**	: **ESI-TRAP**
**Number of queries**	: **130**
**Protein hits**	: gi|120436 Fimbrial protein precursor (Pilin) (Strain PA103)
	gi|1346343 Keratin, type II cytoskeletal 1 (Cytokeratin-1) (CK-1) (Keratin-1) (
	gi|15599752 type 4 fimbrial biogenesis protein PilE [Pseudomonas aeruginosa PAO1
	gi|453155 keratin 9 [Homo sapiens]
	gi|67549 trypsin (EC 3.4.21.4) precursor - bovine

Select Summary Report

Format As | Select Summary (protein hits) | Help

Significance threshold p< 0.05 | Max. number of hits AUTO

Standard scoring ◉ MudPIT scoring ○ | Ions score or expect cut-off 64 | Show sub-sets 0

Show pop-ups ◉ Suppress pop-ups ○ | Sort unassigned Decreasing Score | Require bold red ☑

1. gi|120436 **Mass:** 15789 **Score:** 830 **Queries matched:** 24 **emPAI:** 17.27

Fimbrial protein precursor (Pilin) (Strain PA103)

Query	Observed	Mr(expt)	Mr(calc)	Delta	Miss	Score	Expect	Rank	Peptide
19	591.643636	1181.272720	1182.566940	-1.294220	0	78	0.0022	1	K.FNFATGQSSPK.N 20
43	686.261429	1370.508306	1370.740509	-0.232203	0	(100)	1.3e-05	1	R.SEGASALATINPLK.T
53	707.209474	1412.404396	1412.751068	-0.346672	0	108	2.4e-06	1	R.SEGASALATINPLK.T
78	844.954462	1687.894372	1687.935638	-0.041266	1	88	0.0002	1	K.LGTVAVTIKDTGDGTI
84	657.517027	1969.529253	1969.938156	-0.408903	1	90	0.00015	1	K.DTGDGTIKFNFATGQS
92	1035.087207	2068.159862	2068.057587	0.102275	0	137	3.1e-09	1	K.ILIGTTASTADTTYVG
98	1087.437097	2172.859642	2172.970764	-0.111122	0	90	0.00013	1	R.TAEGVWTCTSTQEEMF
113	1187.613529	2373.212506	2373.238693	-0.026187	1	130	1.4e-08	1	R.SEGASALATINPLKTT
115	794.667220	2380.979832	2381.232574	-0.252742	1	110	1.3e-06	1	K.ILIGTTASTADTTYVG

---

2. gi|1346343 **Mass:** 65978 **Score:** 174 **Queries matched:** 2 **emPAI:** 0.13

Keratin, type II cytoskeletal 1 (Cytokeratin-1) (CK-1) (Keratin-1) (K1) (67 kDa cytokeratin) (Hair

Query	Observed	Mr(expt)	Mr(calc)	Delta	Miss	Score	Expect	Rank	Peptide
37	633.193333	1264.372114	1264.629913	-0.257799	0	64	0.053	1	R.TNAENEFVTIK.K
116	795.304848	2382.892716	2382.944656	-0.051940	0	110	1.4e-06	1	R.GGGGGYGSGGSSYGSG

Proteins matching the same set of peptides:

gi|7428712 **Mass:** 65454 **Score:** 174 **Queries matched:** 2

keratin 1, type II, cytoskeletal - human

gi|11935049 **Mass:** 66027 **Score:** 174 **Queries matched:** 2

keratin 1 [Homo sapiens]

gi|119395750 **Mass:** 65999 **Score:** 174 **Queries matched:** 2

keratin 1 [Homo sapiens]

gi|114644564 **Mass:** 64172 **Score:** 171 **Queries matched:** 2

PREDICTED: similar to keratin 1 [Pan troglodytes]

---

3. gi|15599752 **Mass:** 15270 **Score:** 85 **Queries matched:** 1 **emPAI:** 0.28

type 4 fimbrial biogenesis protein PilE [Pseudomonas aeruginosa PAO1]

Query	Observed	Mr(expt)	Mr(calc)	Delta	Miss	Score	Expect	Rank	Peptide
45	688.698522	1375.382492	1375.640823	-0.258331	0	85	0.00047	1	R.YYSQNPGVGYTK.D

Proteins matching the same set of peptides:

gi|84323198 **Mass:** 13809 **Score:** 85 **Queries matched:** 1
COG4968: Tfp pilus assembly protein PilE [Pseudomonas aeruginosa 2192]

---

4. gi|453155 **Mass:** 61950 **Score:** 66 **Queries matched:** 1 **emPAI:** 0.07
keratin 9 [Homo sapiens]

Query	Observed	Mr(expt)	Mr(calc)	Delta	Miss	Score	Expect	Rank	Peptide
129	1075.100490	3222.279642	3222.274384	0.005258	0	66	0.027	1	R.GGSGGSHGGGSGFGGESG

Proteins matching the same set of peptides:

gi|55956899 **Mass:** 62027 **Score:** 66 **Queries matched:** 1
keratin 9 [Homo sapiens]
gi|81175178 **Mass:** 62092 **Score:** 66 **Queries matched:** 1
Keratin, type I cytoskeletal 9 (Cytokeratin-9) (CK-9) (Keratin-9) (K9)
gi|113197968 **Mass:** 48057 **Score:** 66 **Queries matched:** 1
KRT9 protein [Homo sapiens]
gi|114667176 **Mass:** 107386 **Score:** 66 **Queries matched:** 1
PREDICTED: similar to Keratin, type I cytoskeletal 14 (Cytokeratin-14) (CK-14) (Keratin-14) (K14) [

---

5. gi|67549 **Mass:** 23978 **Score:** 65 **Queries matched:** 1 **emPAI:** 0.18
trypsin (EC 3.4.21.4) precursor - bovine

Query	Observed	Mr(expt)	Mr(calc)	Delta	Miss	Score	Expect	Rank	Peptide
109	758.271463	2271.792561	2272.152130	-0.359569	0	65	0.045	1	K.SIVHPSYNSNTLNNDIML

Proteins matching the same set of peptides:

gi|230338 **Mass:** 23290 **Score:** 65 **Queries matched:** 1
Chain E, Trypsin (E.C.3.4.21.4) Complex With Bowman-Birk Inhibitor (AB-I)
gi|230765 **Mass:** 22871 **Score:** 65 **Queries matched:** 1
Chain E, Bovine Trypsin (E.C.3.4.21.4) Complex With A Modified SSI (Streptomyces Subtilisin Inhibit
gi|1421532 **Mass:** 23975 **Score:** 65 **Queries matched:** 1
Chain , Trypsinogen-Ca From Peg
gi|2392548 **Mass:** 23276 **Score:** 65 **Queries matched:** 1
Chain A, Bovine Trypsin Complexed To Appi
gi|2392803 **Mass:** 23314 **Score:** 65 **Queries matched:** 1
Chain , Structure Of Hydrolase (Serine Proteinase)
gi|2507249 **Mass:** 25408 **Score:** 65 **Queries matched:** 1
Cationic trypsin precursor (Beta-trypsin) [Contains: Alpha-trypsin chain 1; Alpha-trypsin chain 2]
gi|5542503 **Mass:** 24704 **Score:** 65 **Queries matched:** 1
Chain A, Trypsin Inhibitors With Rigid Tripeptidyl Aldehydes
gi|9955040 **Mass:** 23288 **Score:** 65 **Queries matched:** 1
Chain A, Recruiting Zinc To Mediate Potent, Specific Inhibition Of Serine Proteases
gi|13096612 **Mass:** 23879 **Score:** 65 **Queries matched:** 1
Chain A, Bovine Beta-Trypsin Bound To Meta-Amidino Schiff Base Magnesium(Ii) Chelate
gi|34810822 **Mass:** 25392 **Score:** 65 **Queries matched:** 1
Chain B, Non-Covalent Complex Between Alpha-1-Pi-Pittsburgh And S195a Trypsin
gi|49259456 **Mass:** 23288 **Score:** 65 **Queries matched:** 1
Chain T, Benzamidine In Complex With Bovine Trypsin Variant X(Ssri) Bt.C1
gi|49259463 **Mass:** 23318 **Score:** 65 **Queries matched:** 1
Chain T, Trypsin Inhibitor In Complex With Bovine Trypsin Variant X(Sswi)bt.B4
gi|49259467 **Mass:** 23203 **Score:** 65 **Queries matched:** 1
Chain T, Benzamidine In Complex With Bovine Trypsin Varinat X(Ssai) Bt.D1
gi|61873128 **Mass:** 25769 **Score:** 65 **Queries matched:** 1
PREDICTED: similar to pancreas cationic pretrypsinogen isoform 1 [Bos taurus]
gi|88193016 **Mass:** 23287 **Score:** 65 **Queries matched:** 1
Chain E, Crystal Structure Of A Bpti Variant (Cys14->ser) In Complex With Trypsin
gi|88193018 **Mass:** 23402 **Score:** 65 **Queries matched:** 1
Chain E, Crystal Structure Of A Bpti Variant (Cys38->ser) In Complex With Trypsin
gi|49259465 **Mass:** 23321 **Score:** 63 **Queries matched:** 1

Chain T, Benzamidine In Complex With Bovine Trypsin Variant X(Ssfi.Glu)bt.D1
gi|49259461 **Mass:** 23295 **Score:** 62 **Queries matched:** 1
Chain T, Trypsin Inhibitor In Complex With Bovine Trypsin Variant X(Ssyi)bt.B4
gi|112490427 **Mass:** 23134 **Score:** 61 **Queries matched:** 1
Chain A, Structure Of Hyper-Vil-Trypsin

Mascot: http://www.matrixscience.com/

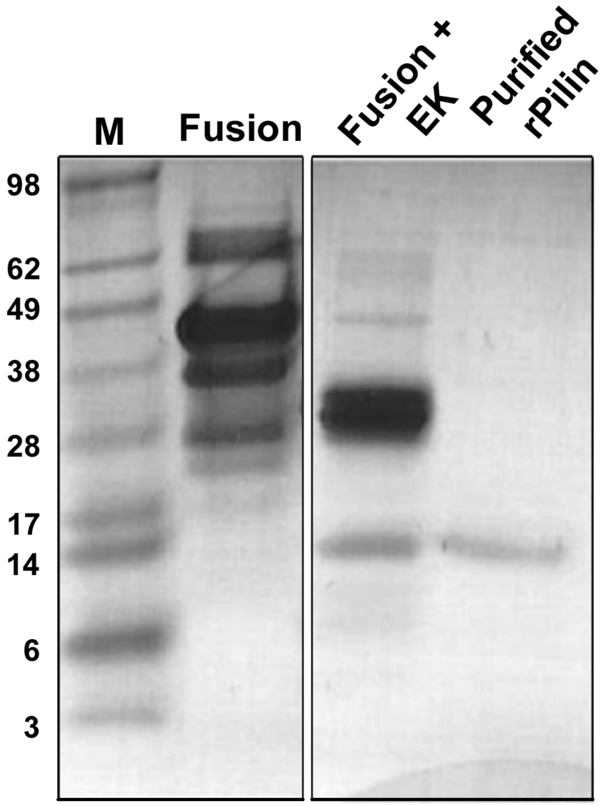

Figure S2

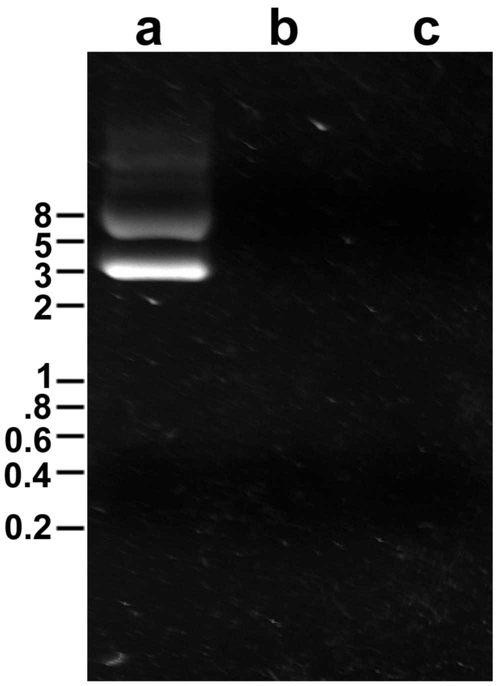

	a	b	c
Plasmid	+	+	+
DNAse I	–	+	+
Pilin	–	–	+

Figure S3

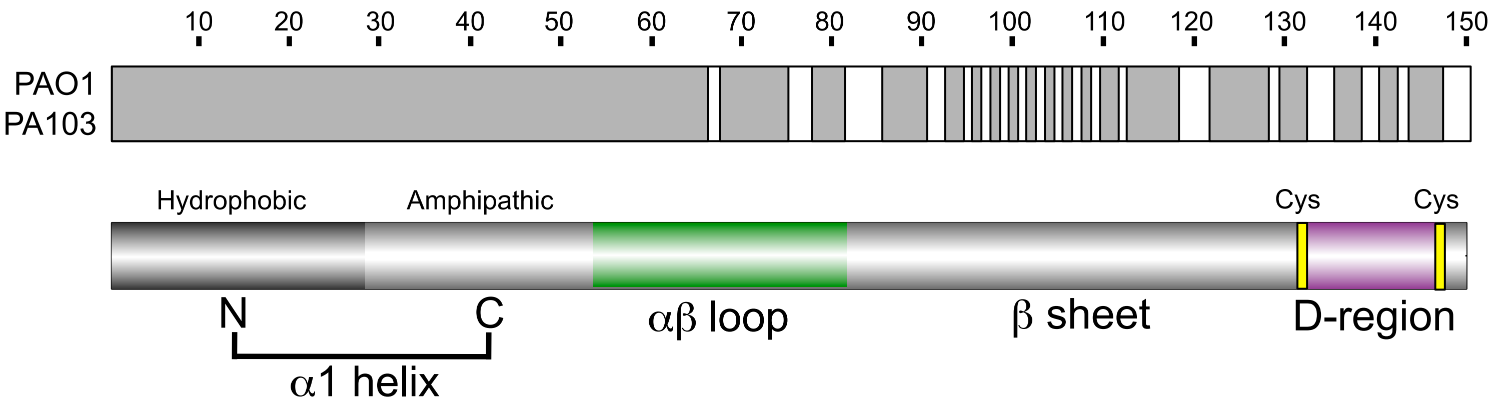
10
20
30
40
50
60
70
80
90
100
110
120
130
140
150
PAO1
PA103
Hydrophobic
Amphipathic
Cys
Cys
N
C
αβ loop
β sheet
D-region
α1 helix

Figure S4.
